# The function of Piezo1 in colon cancer metastasis and its potential regulatory mechanism

**DOI:** 10.1007/s00432-020-03179-w

**Published:** 2020-03-09

**Authors:** Yanhua Sun, Ming Li, Guangjie Liu, Xue Zhang, Lianghui Zhi, Jing Zhao, Guiying Wang

**Affiliations:** 1 Department of General Surgery, The Fourth Hospital of Hebei Medical University, Hebei Cancer Hospital, No. 12 Health Road, Shijiazhuang, 050011 Hebei China; 2 Department of Gastrointestinal Hernia Surgery, Cangzhou People’s Hospital, Cangzhou, Hebei China; 3 Department of Thoracic Surgery, The Fourth Hospital of Hebei Medical University, Hebei Cancer Hospital, Shijiazhuang, Hebei China; 4 Department of Medical Oncology, The Fourth Hospital of Hebei Medical University, Hebei Cancer Hospital, Shijiazhuang, Hebei China; 5 Department of General Surgery, 980th Hospital of Joint Logistic Support Force, Shijiazhuang, Hebei China; 6 Department of Anorectal Surgery, The Second Hospital of Hebei Medical University, Shijiazhuang, Hebei China; 7 Department of General Surgery, Hebei Medical University Third Affiliated Hospital, 139 Ziqiang Road, Shijiazhuang, 050000 Hebei China

**Keywords:** Colon cancer, Metastasis, Piezo1, Mitochondrial calcium uniporter, HIF-1α

## Abstract

**Objective:**

Increasing evidence has revealed that mechanical stress and elevated mechanical signals promote malignant tumor transformation and metastasis. This study aimed to explore the function of the mechanically activated ion-channel Piezo1 in the colon cancer metastasis and its potential regulatory mechanism.

**Methods:**

First, we examined the expression levels of Piezo1 and mitochondrial calcium uniporter (MCU) both in colon cancer tissues and assessed the prognostic value of Piezo1 and MCU in a colon cancer cohort (*n* = 110). Second, functional assays were performed to investigate the effects of Piezo1 and MCU on colon cancer cell migration, invasion, and mitochondrial membrane potential. Third, we analyzed the expression of Piezo1, MCU, and HIF-1α by overexpressing/silencing each other’s expression.

**Results:**

We found that Piezo1 was up-regulated and MCU was down-regulated in colon cancer tissues. Piezo1 and MCU were both correlated with poor prognosis of patients with colon cancer. Overexpressing Piezo1 and silencing MCU could promote colon cancer cell migration and metastasis, reduce mitochondrial membrane potential, and promote each other’s expression. We also found that HIF-1α was up-regulated in colon cancer tissues. Additionally, silencing Piezo1 inhibited the expression of HIF-1α and VEGF, which was contrary to MCU silencing. Intriguingly, Piezo1-overexpressing cells did not regain their migration behaviors when HIF-1α expression was inhibited, which was accompanied with the re-expression of MCU and VEGF.

**Conclusion:**

In our study, Piezo1 is involved in colon cancer cell metastasis. Furthermore, our findings indicated a possible Piezo1-MCU-HIF-1α-VEGF axis, which still need further exploration.

## Introduction

Despite improvements and advances in diagnostic and therapeutic strategies, colon cancer remains the leading cause of cancer-related deaths worldwide (Siegel et al. 2018; Chaffer and Weinberg 2011). Most of these deaths are caused by metastatic diseases (Dong et al. 2019). Surgical resection is the first line of treatment for colon cancer (Li et al. 2019). Most of patients have metastasized during the diagnosis, making surgical resection ineffective. There is no significant improvement in the 5-year survival rate and risk of recurrence in colon cancer patients due to local recurrence and tumor metastasis. Therefore, a deeper understanding of the molecular and cellular basis of metastasis is of high clinical significance (Rokavec et al. 2017). Increasing evidence suggests that many molecular expression changes are involved in signal transduction pathways in colon cancer metastasis by affecting key molecular targets (Zhou et al. 2017; Zykova et al. 2018). Therefore, the identification of key molecular targets is of significant value for the diagnosis and treatment of patients with metastatic colon cancer. In this study, we analyzed a novel mechanically activated ion-channel Piezo1, as a potential molecular target for metastatic colon cancer.

Malignant tumor cells are characterized by migration, invasion, and metastasis (Chubinskiy-Nadezhdin et al. 2019). Tumor cell mobility is affected by a variety of signaling cascades, including ion channels and transporters (Schwab and Stock 2014). Mechanically sensitive calcium-permeable ion channels are mainly involved in the process of cell movement (Chaffer and Weinberg 2011). Mechanically sensitive channels can regulate calcium-dependent signaling cascades associated with tumor cell migration by promoting local calcium influx (Maroto and Hamill 2007). Piezo1, also known as FAM38A, is a member of PIEZO family (including Piezo1 and Piezo2). Coste B et al. first proposed that the Piezo1 protein is a component of a mechanically activated cation channel (Coste et al. 2010, 2012), which directly senses mechanical forces and converts environmental signals into intracellular Ca^2+^ responses (Miyamoto et al. 2014). In addition, Piezo1 has been confirmed to be a cell stretch sensor that integrates physiological forces into vascular structures and is involved in vascular development and function (Li et al. 2014; Ranade et al. 2014). Piezo1 is widely expressed in a variety of cells and tissues, including tumor cells (Liu et al. 2018). Recent research has showed that Piezo1 is involved in TFF1-mediated gastric cancer cell migration (Yang et al. 2014). Furthermore, Piezo1 activated by agonist Yoda1 could promote TRAIL-mediated cell apoptosis via mitochondrial outer membrane permeability (Hope et al. 2019). Except Piezo1, MCU, an evolutionarily conserved Ca^2+^ channel, has been found, which plays a role in regulating intracellular Ca^2+^ signaling for mitochondria (Cui et al. 2017; Ren et al. 2017). HIF-1α, a Ca^2+^-sensitive factor, has been confirmed to be involved in tumor cell metastasis by promoting EMT (Chen et al. 2017). Although Piezo1 has been extensively studied after being confirmed as a mechanical sensor, functional research of this protein is still limited. Therefore, in our study, we explored the regulatory relationships among Piezo1, MCU, and HIF-1α in colon cancer metastasis.

The role of Piezo1 in colon cancer metastasis remains largely unknown. In this study, we provided evidence that Piezo1 was associated with tumor metastasis in colon cancer patients. The expression of Piezo1 was elevated in colon cancer tissues, and the expression of Piezo1 was a prognostic factor for colon cancer patients. Overexpression of Piezo1 promoted colon cancer cell viability, migration, and metastasis. Moreover, we hypothesized Piezo1-MCU-HIF-1α-VEGF axis, a potential regulatory mechanism in colon cancer metastasis.

## Materials and methods

### Human tissue specimens

A cohort of 113 colon cancer patients were enrolled in our study. The colon cancer tissues and corresponding adjacent normal colon tissues were collected from the Fourth Affiliated Hospital of Hebei Medical University (Hebei, China). All patients did not receive chemotherapy before surgery. Colon cancer was independently diagnosed by two pathologists according to the ESMO–ESSO–ESTRO clinical practice guidelines (2013). Every patient signed an informed consent form. Our study was ethically approved by Hebei Medical University Ethics Committee.

### Immunohistochemical analysis

Colon cancer tissue specimens were fixed in 10% buffered formalin and then embedded in paraffin. The sections were serially sectioned at 5 μm, followed by stained with anti‑Piezo1 (1:100; ab128245; Abcam, USA), anti‑MCU (1:150; ab121499; Abcam, USA), and anti-HIF-1α (1:150; bs-0737R, BIOSS, China).

### Cell culture

Two human colon cancer cell lines (HCT-116, SW-480) were purchased from Shanghai Zhong Qiao Xin Zhou Biotechnology Co., Ltd. (Shanghai, China). Cells were maintained in DMEM medium (SH30084.03, Hyclone, USA) containing 10% fetal bovine serum (11,885,084, Gibco, USA) in a humidified atmosphere with 5% CO_2_ at 37 °C.

### Cell transfections

Piezo1-siRNAs and its negative control (NC) siRNA, MCU-siRNAs, and its NC siRNA, Green fluorescent protein (GFP)-PURO-HIF-1α, and its NC were designed and synthesized. According to the manufacturer’s instructions, transfections were performed using Lipofectamine™ 3000 transfection reagent. The target sequences of the siRNAs were as follows: Piezo1-siRNA, sense: 5′-GACUACUUCCUGUUUGAGUCC-3′, antisense: 5′-ACUCAAACAGGAAGUAGUCCC-3′. MCU-siRNA, sense: 5′-CAUAAAGGAGCCAAAAAGUCA-3′, antisense: 5′-ACUUUUUGGCUCCUUUAUGGA-3′. Piezo1 channel activator Yoda1 (21,904, cayman, USA) and MCU agonist Spermine (18,041, cayman, USA) were transfected into HCT-116 and SW-480 cells. After transfection for 48 h, the cells were collected for further experiments.

### Flow cytometry assay

HCT-116, SW-480 cells were seeded into a 6‑well plate (5 × 10^5^ cells/well), treated with different Yoda1 concentrations (50 μM; 100 μM; 200 μM) and incubated for 48 h. The re‑suspended cells were first marked with 400 μl Annexin V‑fluorescein isothiocyanate (FITC) binding buffer (BestBio, Shanghai, China) and digested in 0.25% trypsin without ethylenediaminetetraacetic acid. After that, the cells were centrifuged at 1000 × *g* at room temperature for 5 min and washed three times with cold PBS. After incubation with Annexin V‑FITC (5 μl) for 20 min, propidium iodide (10 μl) was added and incubated for 5 min at 4 ˚C. The stained cells were immediately analyzed by flow cytometry (BD Biosciences, San Jose, CA, USA).

### Quantitative real-time polymerase chain reaction (qRT-PCR)

Total RNA was extracted using Trizol reagent (15,596–026, Invitrogen, USA) from tumor tissues and cells. Afterwards, total RNA was reverse transcribed into cDNA using a TaqMan real-time PCR kit (TaqMan). qRT-PCR was carried out on a Bio-Rad CFX96 real-time PCR detection system (CFX96, Bio-Rad, USA). The primers were used to amplify the target genes. GADPH served as an internal control. The relative expression levels were calculated by the comparative 2^–ΔΔCt^ method. The sequences of primers are listed in Table 1.

**Table 1:** Primers of target genes for qRT-PCR

Target genes	Primer sequence (5′–3′)
Piezo 1	F: 5′-GGACTCTCGCTGGTCTACCT-3′
	R: 5′-GGGCACAATATGCAGGCAGA-3′
MCU	F: 5′-GTTTCCAGTTGAGAGATGGCG-3′
	R: 5′-ATTCTGCCAGGAAGCGATCC-3′
HIF-1α	F: 5′-GAAAGCGCAAGTCTTCAAAG-3′
	R: 5′-TGGGTAGGAGATGGAGATGC-3′
VEGF	F: 5′-CTCGCAGTCGCGGAGA-3′
	R: 5′-GCAGCCTGGACCCTTGGC-3′
GADPH	F: 5′-CAAGGTCATCCATGACAACTTTG-3′
	R: 5′-GTCCACCACCCTGTTGCTGTAG-3′

### Western blotting

Total protein was extracted from tumor tissues and cells using RIPA buffer supplemented with proteinase inhibitor. After that, total protein was separated using SDS-PAGE and then transferred onto a PVDF membrane (IPVH00010; Millipore, USA). The membranes were blocked using 5% solution non-fat milk for 1 h. After incubation with primary antibodies at 4 °C overnight, the membranes were incubated with secondary antibody for 1 h at room temperature. The target proteins were visualized using BeyoECL Plus (KGP1121; Nanjing KeyGen Biotech Co., Ltd.) method. The primary antibodies used were as follows: anti‑Piezo1 (1:1000; ab128245; Abcam, USA), anti‑MCU (1:1000; ab121499; Abcam, USA), anti- HIF-1α (1:1000; bs-0737R; BIOSS, China), anti-VEGF (1:1000; bs-0279R; BIOSS, China), and β-actin (1:1000; bs-0061R; BIOSS, China). β-actin was performed as an endogenous control.

### Cell counting kit-8 (CCK-8)

Cultured cells were seeded into 96-well plates in triplicate. The cell proliferation was tested with CCK-8 assay. At 48 h after transfection, CCK-8 (Dojindo Molecular Technologies, Inc., Kumamoto, Japan) was added into the cultured cells. The absorbance at 450 nm was examined with a multiscan spectrum.

### Cell migration assay

Cell migration was performed in 24-well transwell chambers (3412; Corning, USA). The upper chambers were uncoated with Matrigel. The transfected cells were added onto the upper chamber and cultured for 48 h. The transwell membranes were fixed and stained with 0.1% crystal violet for 2 min. Under an optical microscope, cells adhering to the lower surface of the membrane were counted (IX71; OLYMPUS, Japan).

### Wound-healing assay

Transfected cells were plated into 6-well plates. Cells were grown to a confluence of 100% allowed to wound-healing assay. The confluent cells were scratched with a 200 μm-pipette tip. The wound closure was imaged under a microscope at 0 and 48 h. The wound closure width was measured using ImageJ software.

### Immunocytochemical analyses

Transfected cells were seeded on the culture dish (30 × 10^4^/dish). Then, the cells were fixed in 4% paraformaldehyde (PFA) for 15 min and incubated in hydrogen peroxide for 15 min. The cells were blocked with goat serum (Abcam, USA) at 37 °C for 30 min, followed by incubation with anti‑Piezo1 (1:100; ab128245; Abcam, USA), anti‑MCU (1:150; ab121499; Abcam, USA), and anti-HIF-1α (1:150; bs-0737R, BIOSS, China).

### Mitochondrial membrane potential (MMP)

Cells were seeded into 6-well plates containing serum and phenol red. Then, the cells were treated by test compounds for 24 h. After that, the cells were stained with JC-1 dye (M8650, Solarbio, China). The mitochondrial uncoupler carbonyl cyanide 3-chlorophenylhydrazone (CCCP) served as a positive control. The results were observed under a fluorescence microscope (IX71; OLYMPUS, Japan). The level of mitochondrial membrane potential was quantified by Image-Pro Plus software (IPP 6.0, USA).

### Statistical analyses

The data were analyzed by Graphpad 7.0 and SPSS 18.0. All quantitative data are expressed as the means ± standard deviation (SD). The differences between two groups were compared by Student’s *t* test. Furthermore, one-way analysis of variance was used for multiple comparisons. The correlations between Piezo1 expression and clinical parameters were evaluated. Kaplan–Meier method was used to estimate the survival rate for the expression of Piezo1 and MCU, and the survival curves were examined by log-rank tests. *p* < 0.05 was considered statistically significant.

## Results

### Piezo1 is up-regulated in colon cancer tissues and correlated with the prognosis of colon cancer patients

**Fig. 1:**
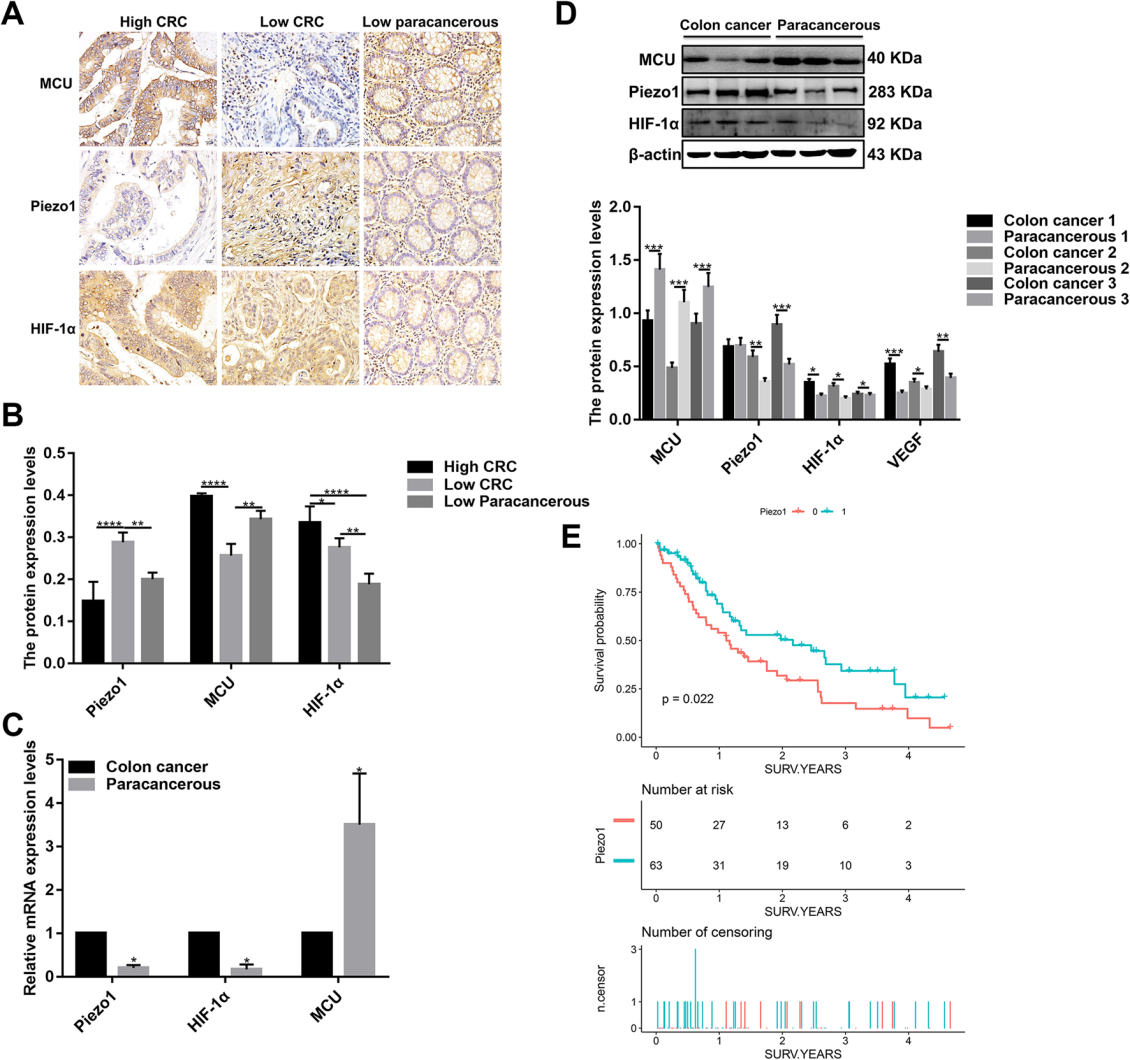
The expression of Piezo1, MCU, and HIF-1α in colon cancer tissues, and Piezo1 expression was in association with colon cancer patients’ prognosis. **a** Representative images of immunohistochemistry for high- or low-differentiation colon cancer tissues and adjacent normal tissues. **b** The expression of Piezo1, MCU, and HIF-1α in high- or low-differentiation colon cancer tissues and adjacent normal tissues according to immunohistochemistry results. **c** qRT-PCR analysis of the expression of Piezo1, MCU, and HIF-1α in colon cancer tissues and corresponding adjacent normal tissues. **d** Western blot analysis of the expression of Piezo1, MCU, and HIF-1α in colon cancer tissues and adjacent normal tissues. **e** Kaplan–Meier survival analysis showed that the expression of Piezo1 was correlated with colon cancer patients’ overall survival (*p* = 0.022). Each experiment was repeated ≥ 3. *p < 0.05; **p < 0.01; ***p < 0.001; ****p < 0.0001

To determine whether Piezo1 participated in the development of colon cancer, we first examined the expression of Piezo1 by immunohistochemistry. The result showed that Piezo1 expression was higher in most of low-differentiation colon adenocarcinoma tissues compared to high-differentiation colon adenocarcinoma tissues and adjacent normal low-differentiation colon tissues (Fig. 1a, b). To further investigate our findings, we measured Piezo1 expression in three low-differentiation colon adenocarcinoma tissues. At the mRNA and protein levels, Piezo1 expression was higher in colon cancer tissues than in the adjacent normal colon tissues (Fig. 1c, d).

113 colon cancer patients were divided into two groups including Piezo1 high-expression group and Piezo1 low-expression group according to the median value of Piezo1 expression. Survival analysis showed that patients with high Piezo1 expression had shorten overall survival rate compared to those with low Piezo1 expression (Fig. 1e). Furthermore, to evaluate the clinical significance of Piezo1 in colon cancer, the correlation between its expression level and clinical parameters was analyzed. As shown in Table 2, Piezo1 expression levels were significantly correlated with vascular invasion (*p* = 0.022).

**Table 2:** Association between clinical parameters and Piezo1 expression in colon cancer

	Piezo1 high expression (*n* = 50)	Piezo1 low expression (*n* = 63)	*p *value
Age > 60 years	22 (44.00%)	31 (49.21%)	0.581
Male	29 (58.00%)	27 (42.86%)	0.110
TNM stage			0.148
1	6 (12.00%)	5 (7.94%)	
2	4 (8.00%)	10 (15.87%)	
3	13 (26.00%)	25 (39.68%)	
4	27 (54.00%)	23 (36.51%)	
T			0.598
1	2 (4.00%)	1 (1.59%)	
2	6 (12.00%)	10 (15.87%)	
3	25 (50.00%)	36 (57.14%)	
4	17 (34.00%)	16 (25.40%)	
N			0.985
0	15 (30.00%)	19 (30.16%)	
1	35 (70.00%)	44 (69.84%)	
M			0.160
0	26 (52.00%)	41 (65.08%)	
1	24 (48.00%)	22 (34.92%)	
Size > 5 cm	39 (81.25%)	53 (84.13%)	0.690
Follow up (years)	1.40 ± 1.20	1.47 ± 1.25	0.743
Survival rate	9 (18.00%)	32 (50.79%)	< 0.001***

***Indicates *p* < 0.001

### Effects of Piezo1 on cell viability and MMP in colon cancer cells

**Fig. 2:**
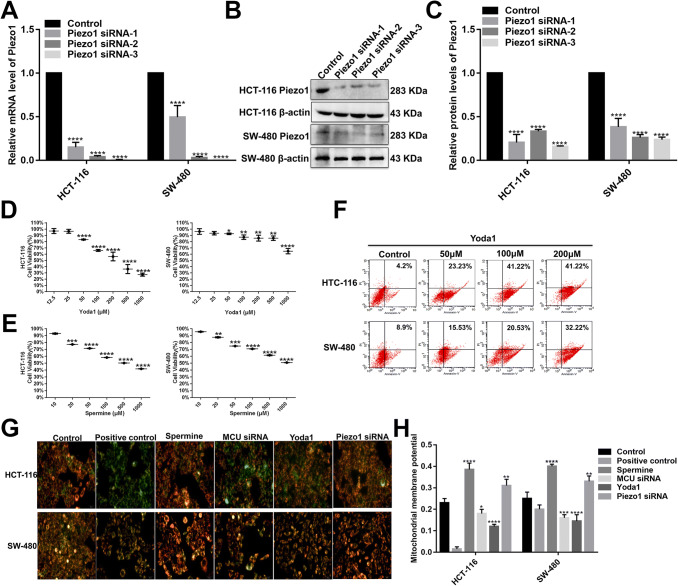
The effects of Piezo1 and MCU on cell viability and MMP in colon cancer cells. **a**–**c** Three specific siRNAs were used for silencing Piezo1 in HCT-116 and SW-480 cells. qRT-PCR and western blotting analysis results revealed that Piezo1 expression was stably inhibited by three Piezo1-siRNAs. CCK-8 assay was performed to investigate the cell viability in HCT-116 and SW-480 cells after transfection with Piezo1 channel activator Yoda1 (**d**) and MCU agonist Spermine (**d**). **f** Flow cytometry assay was performed to evaluate the cell apoptosis of HCT-116 and SW-480 cells transfected with Yoda1. **g**, **h** Mitochondrial membrane potential was measured following knockdown or overexpression of Piezo1 and MCU. Each experiment was repeated ≥ 3. *p < 0.05; **p < 0.01; ***p < 0.001; ****p < 0.0001

We further observed the effects of Piezo1 overexpression and loss on cellular biological functions. We chose two colon cancer cell lines (HCT-116 and SW-480). Three specific siRNAs were used for silencing Piezo1. To examine the transfected effects in HCT-116 and SW-480 cells, qRT-PCR and western blotting analyses were performed (Fig. 2a–c). The results revealed that the Piezo1-siRNA-3 stably inhibited Piezo1 expression, which was used for further experiments.

Piezo1 overexpression was achieved by Piezo1 channel activator Yoda1. Yoda1 has been confirmed as a specific tool to activate Piezo1 ion channel in a variety of cells and tissues (Tsuchiya et al. 2018). We examined the effect of Yoda1 with different concentrations on the cell viability of HCT-116 and SW-480 cell lines using CCK-8 assay (Fig. 2d, e). Apoptotic cells were marked with Annexin V/PI and analyzed via flow cytometry (Fig. 2f). The results showed that cell viability was inhibited and apoptosis was promoted in a dose-dependent manner. The optimal concentration of Yoda1 (100 μM) was determined for further experiments. The mitochondrion-specific dye JC-1 was used to observe whether Piezo1 affected the MMP in HCT-116 and SW-480 cells. We found that Piezo1 overexpression dissipated the MMP compared to Piezo1 knockdown (Fig. 2g, h), indicating that Piezo1 could mediate colon cancer cell apoptosis.

### Piezo1 promotes colon cancer cell metastasis

**Fig. 3:**
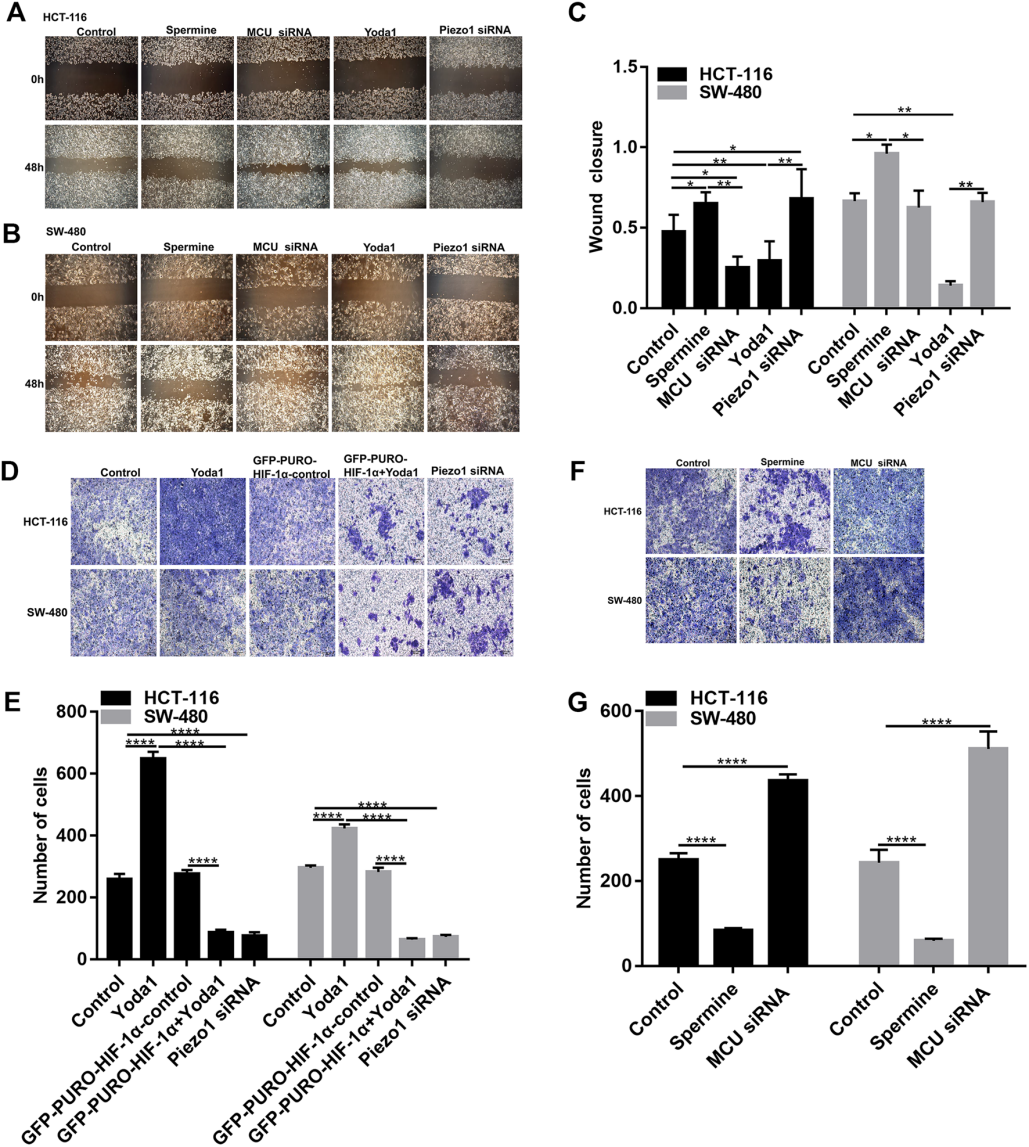
The effect of Piezo1 and MCU in colon cancer cell metastasis. **a**–**c** Wound-healing assay was performed to investigate the effect of Piezo1 and MCU on cell motility in HCT-116 and SW-480 cells. **d**–**g** Transwell assay was used to evaluate the effect of Piezo1 and MCU on cell migration. *p < 0.05; **p < 0.01; ****p < 0.0001

Transwell and wound-healing assays were performed to investigate whether Piezo1 was in association with colon cancer metastasis. Wound-healing assay results showed that wound closure was slower in Piezo1-knockdown HCT-116 and SW-480 cells than in controls (Fig. 3a–c); however, Piezo1-overexpressing cells migrated faster to the scratch area than controls. Similarly, transwell assay results suggested that the cell migration was inhibited in Piezo1-silenced HCT-116 and SW-480 cells compared to controls, while cell migration was promoted in HCT-116 and SW-480 cells with Piezo1 channel activator Yoda1 compared to controls (Fig. 3d, e). These results indicate that Piezo1 promotes colon cancer cell metastasis in vitro.

### MCU is down-regulated in colon cancer tissues and associated with cell viability, MMP, and metastasis in colon cancer cells

**Fig. 4:**
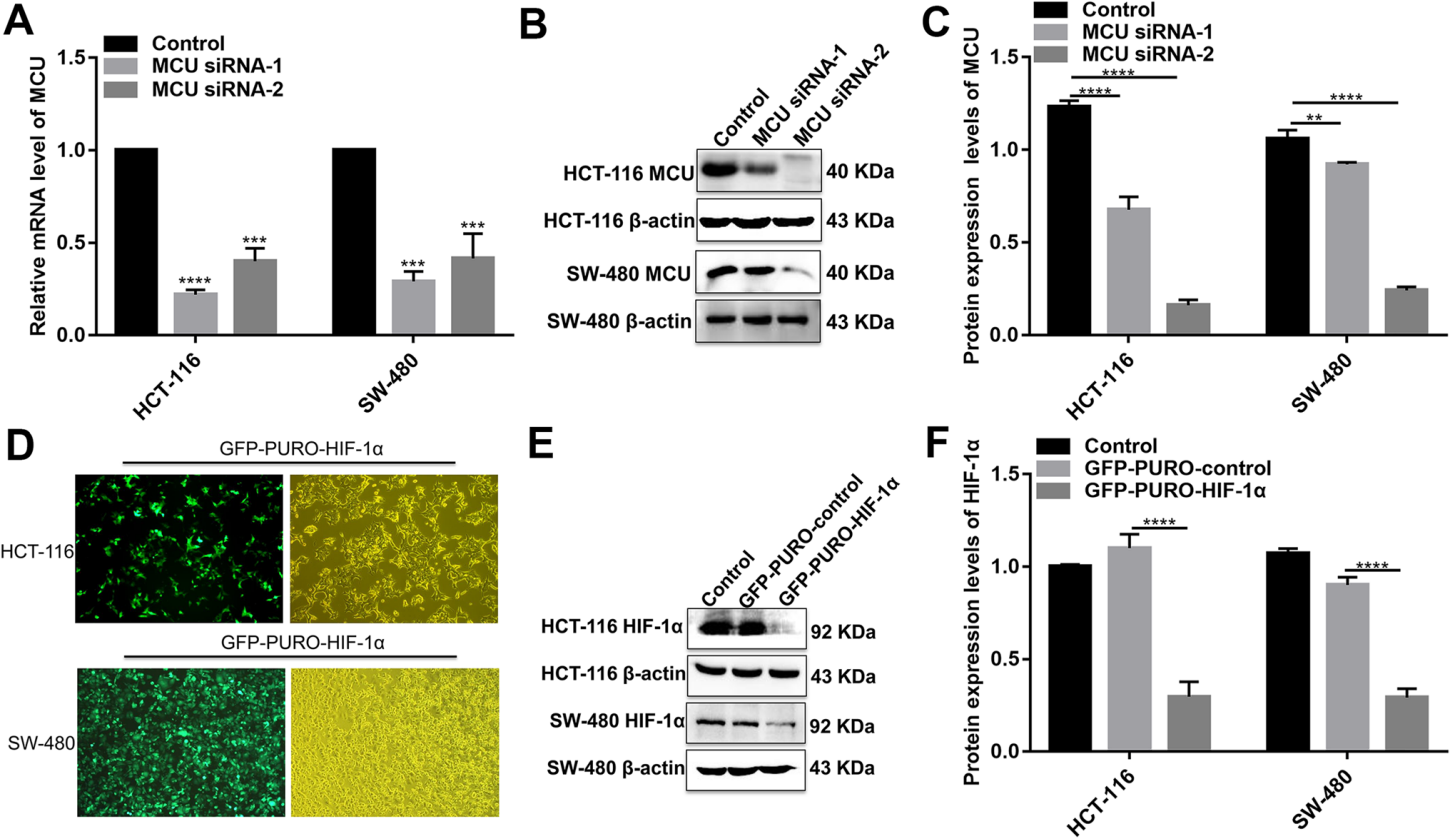
The expression of MCU and HIF-1α was successfully silenced in HCT-116 and SW-480 cells. **a**–**c** qRT-PCR and western blotting analysis results showed that MCU expression was silenced by two MCU-siRNAs in HCT-116 and SW-480 cells. **d** Representative immunofluorescence images showing HIF-1α expression in HCT-116 and SW-480 cells transfected by GFP-PURO-HIF-1α. **e**, **f** Western blotting analysis showing HIF-1α expression following HIF-1α knockdown in HCT-116 and SW-480 cells. Each experiment was repeated ≥ 3. **p < 0.01; ***p < 0.001; ****p < 0.0001

MCU expression was lower in high- or low-differentiation colon cancer tissues than adjacent normal tissues (Fig. 1a, b), which was confirmed by qRT-PCR and western blotting analysis (Fig. 1b–d). MCU was silenced with two specific siRNAs. qRT-PCR and western blotting analysis results showed that MCU was significantly inhibited in HCT-116 and SW-480 cells (Fig. 4a–c). CCK-8 assay results suggested that cell viability was inhibited for HCT-116 and SW-480 cells with MCU agonist Spermine, in a dose-dependent manner (Fig. 2e). JC-1 assay revealed that MCU knockdown reduced MMP compared to MCU overexpression in two colon cancer cells (Fig. 2g, h). Wound-healing assays showed that MCU-overexpressing cells migrated slower to the scratch area than controls; however, the opposite result was observed when MCU was suppressed (Fig. 3a–c). In addition, migration assay demonstrated that more cells invaded through the Matrigel layer when MCU was silenced, which was contrary to MCU-overexpressed cells (Fig. 3f, g).

**Fig. 5:**
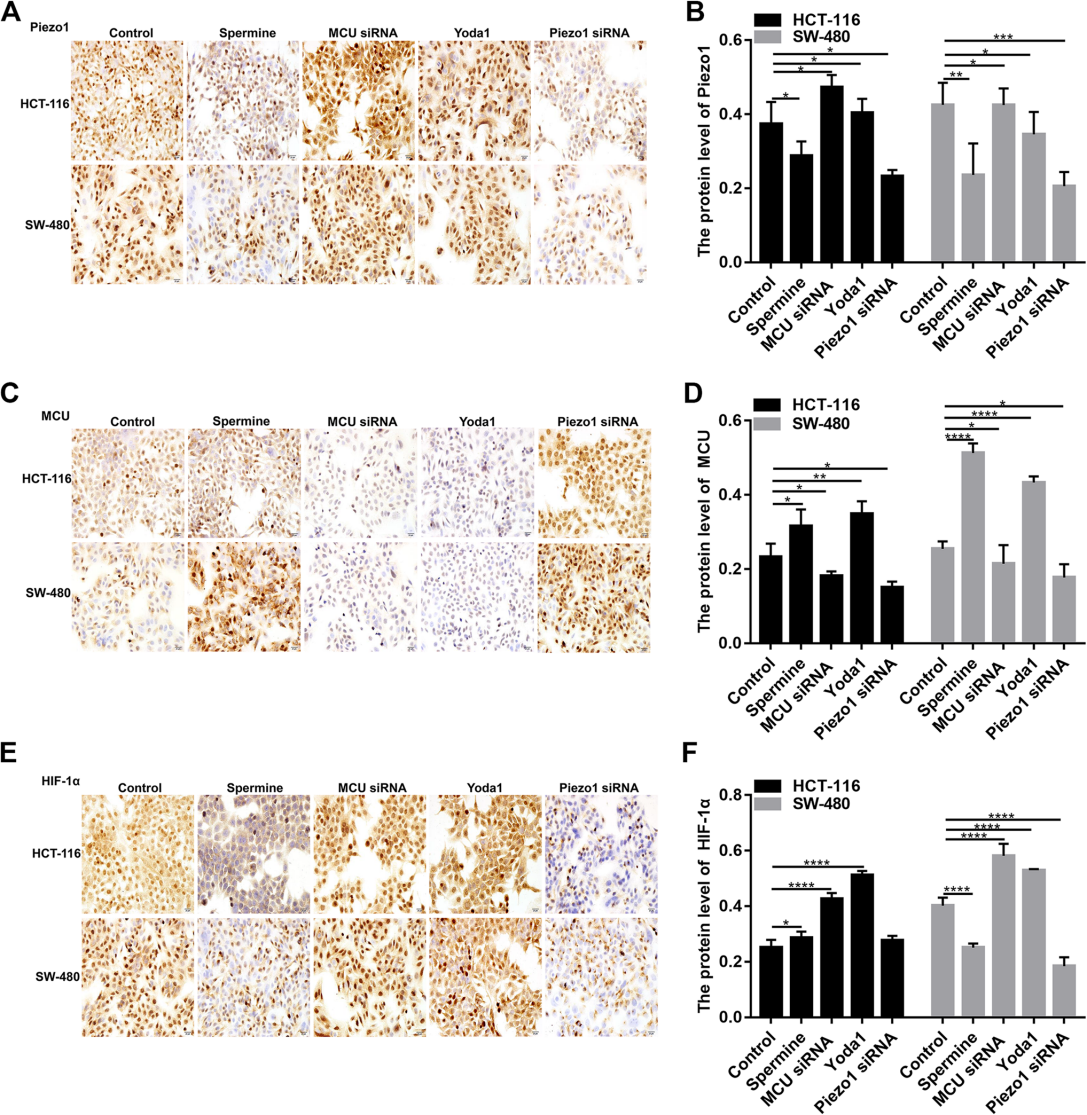
The expression of Piezo1, MCU and HIF-1α following overexpression or knockdown of Piezo1 and MCU in HCT-116 and SW-480 cells. Immunohistochemistry results showing the expression of Piezo1 (**a**, **b**), MCU (**c**, **d**), and HIF-1α (**e**, **f**) in HCT-116 and SW-480 cells. Each experiment was repeated ≥ 3. *p < 0.05; **p < 0.01; ***p < 0.001; ****p < 0.0001

In HCT-116 and SW-480 cells, immunohistochemistry results found that Piezo1 expression was significantly higher in MCU-silenced group than in MCU-overexpressed group (Fig. 5a, b). In addition, MCU expression was obviously promoted in Piezo1-overexpressed group compared to Piezo1-silenced group (Fig. 5c, d). Above results indicate that MCU could be a downstream target of Piezo1.

### Piezo1 could promote cell metastasis through MCU targeting HIF-1α

**Fig. 6:**
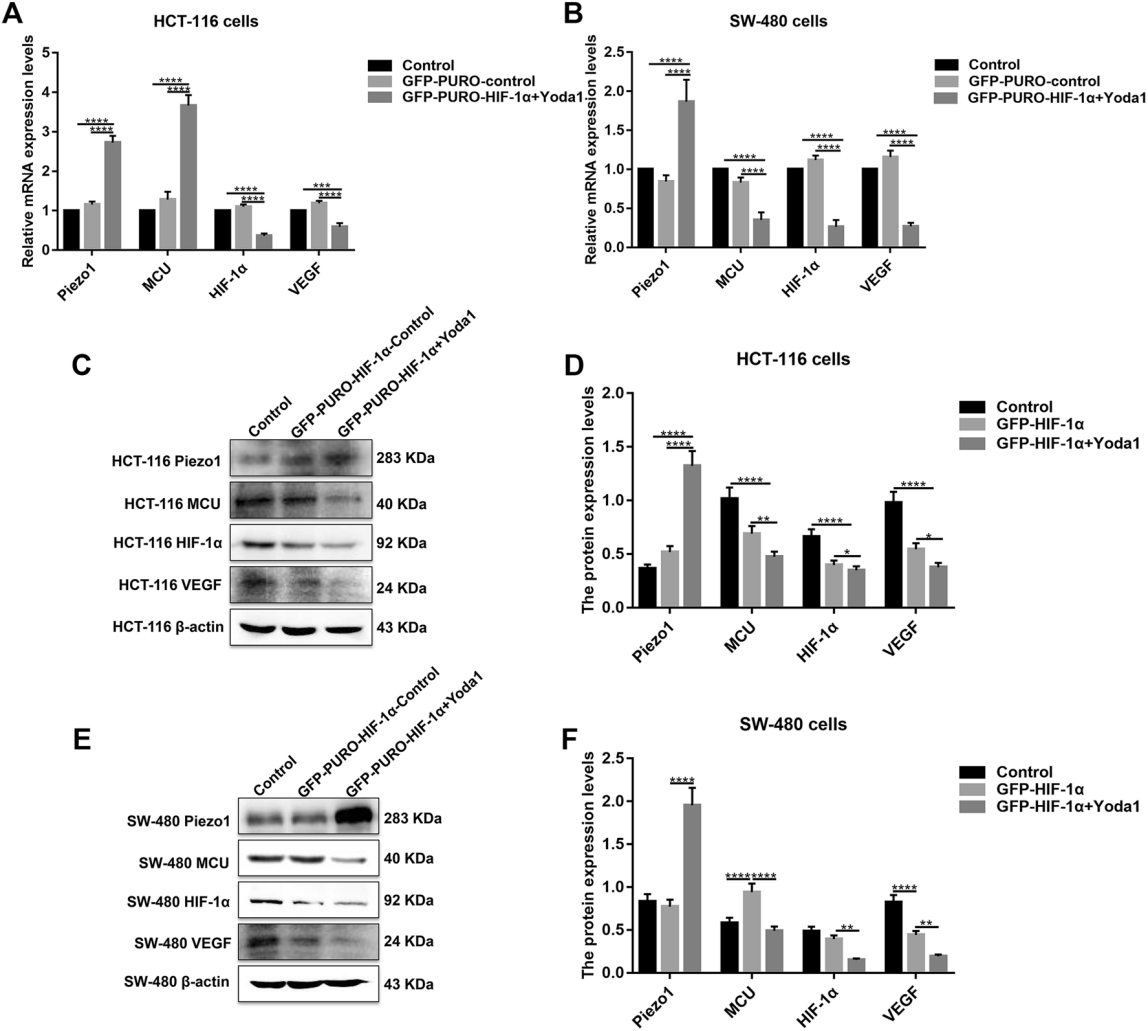
The expression of Piezo1, MCU, HIF-1α, and VEGF following Piezo1 overexpression and HIF-1α knockdown in HCT-116 and SW-480 cells. **a**, **b** qRT-PCR results showing the mRNA expression of Piezo1, MCU, HIF-1α, and VEGF in HCT-116 and SW-480 cells. **c**–**f** Western blotting analysis showing the protein expression of Piezo1, MCU, HIF-1α, and VEGF in HCT-116 and SW-480 cells. Each experiment was repeated ≥ 3. *p < 0.05; **p < 0.01; ***p < 0.001; ****p < 0.0001

HIF-1α was up-regulated in high or low-differentiation colon cancer tissues compared to adjacent normal tissues (Fig. 1a, b), which was verified by qRT-PCR and western blots (Fig. 1c, d). To investigate the role of HIF-1α in colon cancer, HIF-1α was successfully silenced according to immunofluorescence and western blots (Fig. 4d–f). Transwell assay results showed that, in GFP-PURO-HIF-1α + Yoda1 group, cell migrated ability was significantly reduced compared to Yoda1 group and GFP-PURO-HIF-1α-Control (Fig. 3d, e), which was suggesting that when HIF-1α expression was inhibited, overexpressed Piezo1 lost the role of promoting colon cancer cell migration. Additionally, immunohistochemistry results showed that HIF-1α expression was elevated after Piezo1 overexpression or MCU knockdown in HCT-116 and SW-480 cells (Fig. 5e, f). In GFP-PURO-HIF-1α + Yoda1 group, the expression of Piezo1 and MCU was higher than controls, while HIF-1α expression was down-regulated at the mRNA and protein levels (Fig. 6a–f). These results indicated that HIF-1α could be a downstream of Piezo1 and MCU, and Piezo1 could promote cell migration through MCU targeting HIF-1α.

### Piezo1-MCU-HIF-1α-VEGF: a possible signaling pathway in colon cancer metastasis

**Fig. 7:**
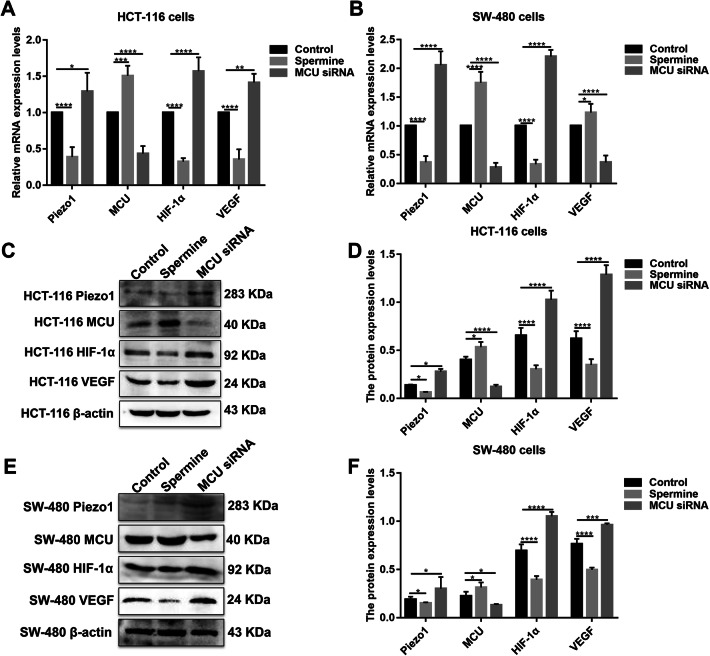
The expression of Piezo1, MCU, HIF-1α, and VEGF following MCU overexpression or knockdown in HCT-116 and SW-480 cells. **a**, **b** qRT-PCR showing the mRNA expression of Piezo1, MCU, HIF-1α, and VEGF in HCT-116 and SW-480 cells. **c**–**f** Western blotting analysis showing the protein expression of Piezo1, MCU, HIF-1α, and VEGF in HCT-116 and SW-480 cells. Each experiment was repeated ≥ 3. *p < 0.05; **p < 0.01; ***p < 0.001; ****p < 0.0001

**Fig. 8:**
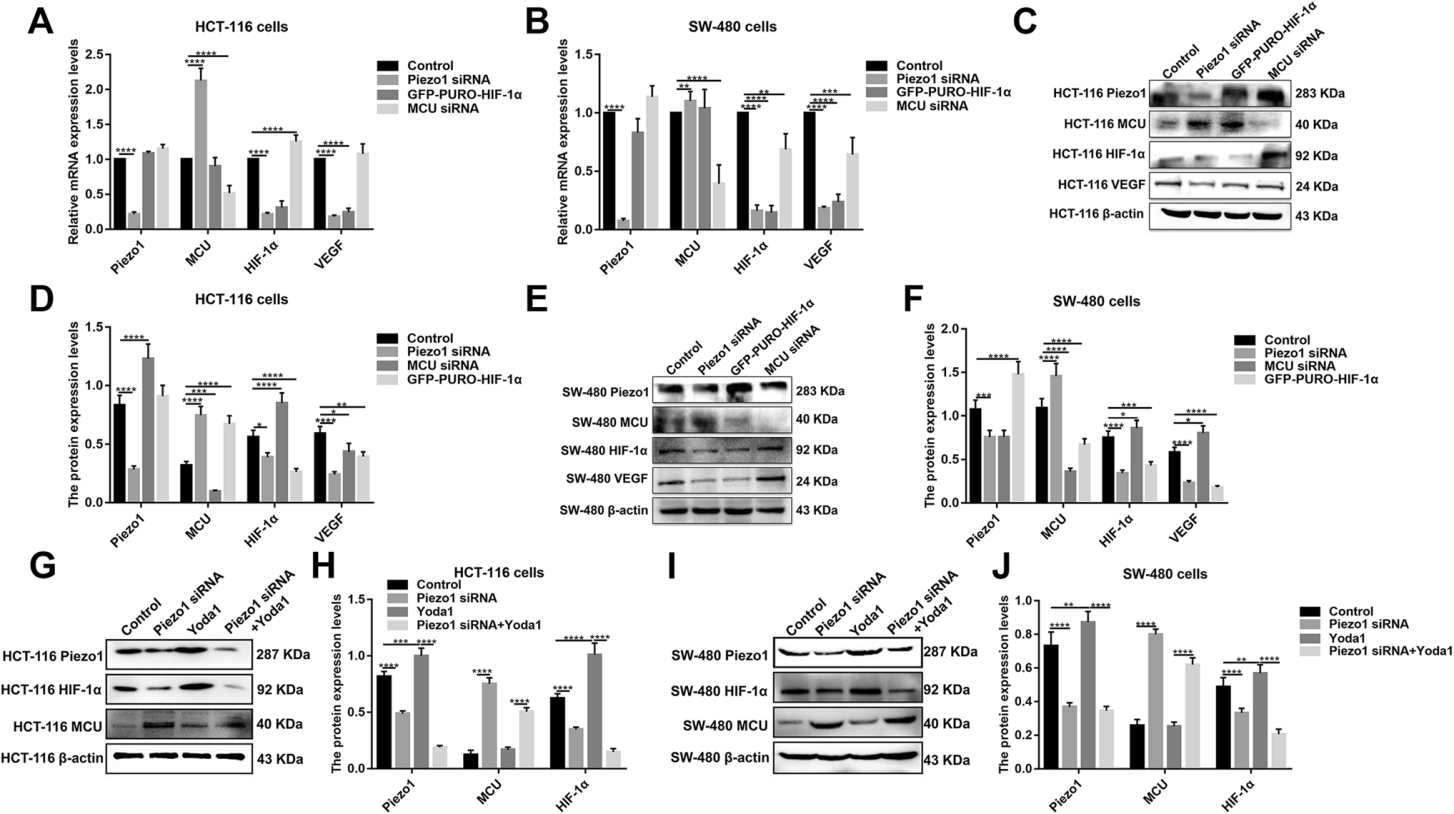
The expression of Piezo1, MCU, HIF-1α, and VEGF in HCT-116 and SW-480 cells transfected by knockdown of Piezo1, HIF-1α, or MCU. **a**, **b** qRT-PCR analysis showing the mRNA expression of Piezo1, MCU, HIF-1α, and VEGF in HCT-116 and SW-480 cells. **c**–**f** Western blotting analysis showing the protein expression of Piezo1, MCU, HIF-1α, and VEGF in HCT-116 and SW-480 cells. **g**–**j** Western blotting analysis showing the protein expression of Piezo1, MCU, and HIF-1α in HCT-116 and SW-480 cells transfected with Piezo1 knockdown or overexpression. Each experiment was repeated ≥ 3. *p < 0.05; **p < 0.01; ***p < 0.001; ****p < 0.0001

**Fig. 9:**
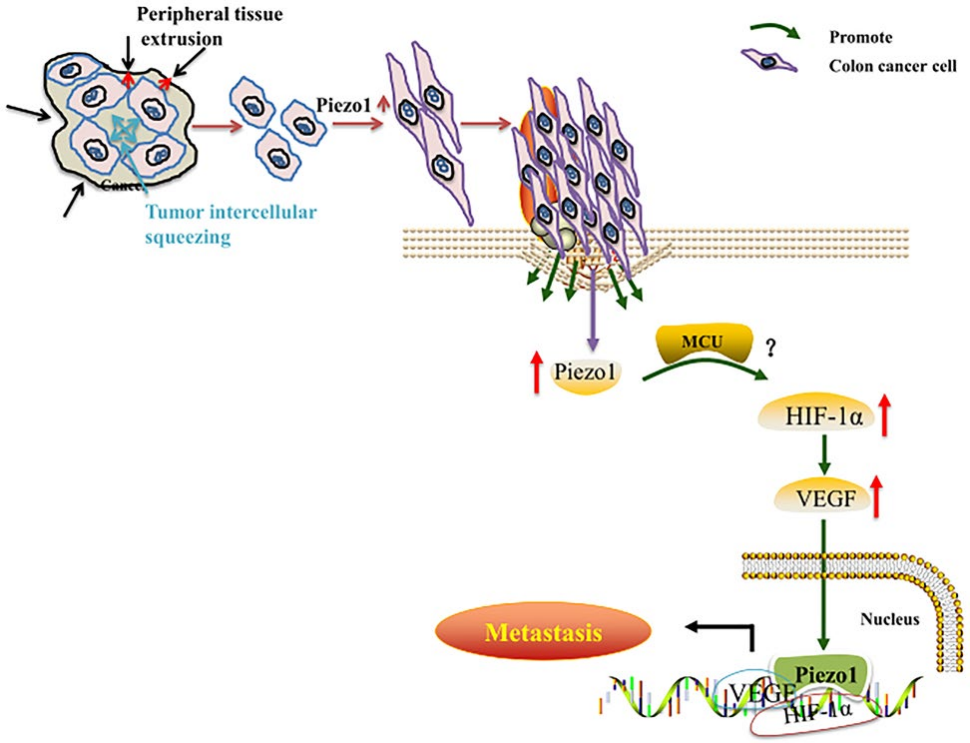
The mechanism diagram in this study

VEGF is involved in the migration, invasion, and metastasis processes of tumor cells. VEGF has been confirmed as a downstream target activated by HIF-1 (Lee et al. 2018). In our study, after inhibiting HIF-1α and overexpressing Piezo1, VEGF expression was slightly decreased (Fig. 6a–f). However, after inhibiting MCU, VEGF expression pattern was different in HCT-116 and SW-480 cells (Fig. 7a–f). The results gave evidence that VEGF was a downstream target gene of HIF-1α. Intriguingly, we found that, after silencing MCU, the expression of Piezo1, HIF-1α was significantly elevated (Fig. 7a–f). After inhibiting Piezo1 expression, MCU expression was elevated and HIF-1α was inhibited, at the mRNA and protein levels (Fig. 8a–f). When HIF-1α was silenced, its downstream target VEGF expression was elevated (Fig. 8a–f); however, Piezo1 expression had no significant change (Fig. 8a–f). More importantly, we validated the activation effect of Yoda1 on Piezo1 in HCT-116 and SW-480 cells. The results showed that Yoda1 significantly elevated the expression of Piezo1; furthermore, Yoda1 significantly promoted the expression of MCU and HIF-1α (Fig. 8g–j). After knockdown of Piezo1, Yoda1 was significantly inhibited. Moreover, we detected the expression of MCU and HIF-1α after Piezo1 knockdown plus Yoda1 treatment. We found that Piezo1 knockdown plus Yoda1 treatment significantly reversed the decreased expression of MCU and HIF-1α induced by Yoda1, determining that Yoda1 was acting through Piezo1 not other potential targets (Fig. 8g–j). These results strongly indicated a possible signaling pathway, Piezo1-MCU-HIF-1α-VEGF in colon cancer cell metastasis (Fig. 9). However, the specific mechanism still needs further exploration.

## Discussion

Despite extensive functional studies, the potential role of Piezo1 in colon cancer metastasis remains largely unclear. In this study, we found that Piezo1 expression was up-regulated in colon cancer tissues. With regard to the mechanism by which Piezo1 is up-regulated in colon cancer, we found that Piezo1 mRNA was significantly increased. Furthermore, high Piezo1 expression was associated with vascular invasion and shorten survival time, indicating that Piezo1 could be associated with poor prognosis. Piezo1 has been confirmed to be associated with prognosis of several cancers, like breast cancer, glioma, and non-small cell lung cancer (Chen et al. 2018; Huang et al. 2019; Li et al. 2015).

Piezo1 was activated by Yoda1 that is an effective activator of Piezo1 channel (Syeda et al. 2015), and highly expressed Piezo1 in HCT-116 and SW-480 cells was in negative association with cell viability; however, silencing Piezo1 suppressed colon cancer cell viability. Moreover, activated Piezo1 reduced MMP, suggesting that Piezo1 was associated with tumor cell death. Thereby, Piezo1 could participate in colon cancer progression by reducing cell viability. It has been found that Piezo1 could regulate synovial sarcoma cell viability (Suzuki et al. 2018). The most important biological feature of malignant tumors, including colon cancer, is the invasion and metastasis of tumor cells. Piezo1 is involved in gastric cancer cell migration and invasion (Yang et al. 2014; Zhang et al. 2018). Our findings revealed that overexpressed Piezo1 promoted colon cancer cell migration and motility. Tumor metastasis is a very complex biological process in which the formation of tumor blood vessels plays an important nutritional support role. Vascular endothelial growth factor (VEGF) is a cell regulatory factor that affects tumor angiogenesis and is closely related to tumor invasion, metastasis, and clinical pathological prognosis (Pang et al. 2018). Previous research found that knockdown of Piezo1 attenuated migration of human umbilical vein endothelial cells towards VEGF (Li et al. 2014). In the present study, VEGF expression was inhibited after overexpression of Piezo1, suggesting that Piezo1 could mediate VEGF expression, thereby affecting colon cancer cell metastasis. Taken together, Piezo1 is involved in colon cancer cell viability, apoptosis, migration, and metastasis.

Mitochondrial dysfunction is closely related to tumorigenesis. Ca^2+^ regulates a variety of essential cellular processes, including processes associated with tumor metastasis (Berridge et al. 2000; Rizzuto et al. 2003). Mitochondria regulate intracellular Ca^2+^ signaling and are responsible for the rapid uptake of Ca^2+^, which are primarily mediated by MCU (Cui et al. 2017; Ren et al. 2017). MCU is an evolutionarily conserved Ca^2+^ channel that is widely expressed and localized on the mitochondrial inner membrane (Vultur et al. 2018). Therefore, MCU plays an important role in regulating aerobic metabolism and cell survival. The role of MCU in cell death and tumorigenesis remains controversial (Zeng et al. 2018). In the present study, we found that MCU was down-regulated in colon cancer tissues. Contrary to Piezo1, MCU knockdown promoted colon cancer cell viability, migration, motility, and metastasis. When silencing Piezo1 or MCU, each other’s expression was elevated in HCT-116 and SW-480 cells. Our study indicated MCU could be a downstream target of Piezo1. However, specific regulatory mechanism remains unclear.

HIF-1α is a Ca^2+^-sensitive factor that promotes EMT by directly regulating Twist expression (Chen et al. 2017). Hypoxia in the tumor followed by HIF-1α is one of the most important mechanisms to promote tumor progression and metastasis (Yang and Wu 2008). Consistent with previous studies, in the present study, HIF-1α expression was up-regulated in colon cancer tissues. In colon cancer cells treated with Yoda1, HIF-1α expression was significantly up-regulated, whereas HIF-1α expression was significantly inhibited after silencing Piezo1. Furthermore, overexpressed Piezo1 significantly promoted colon cancer cell migration. However, the cell migration and VEGF expression was significantly inhibited in HCT-116 and SW-480 cells with overexpressed Piezo1 and knockdown HIF-1α. Taken together, our findings suggested that HIF-1α could be a downstream target of Piezo1 and mediated Piezo1-independent cell migration. Previous study reported that MCU overexpression is in association with poor prognosis of breast cancer. MCU knockdown inhibits HIF-1α expression, thereby attenuating the transcription of HIF-1α involved in tumor progression (Tang et al. 2015). Rescue of HIF-1α expression restores MCU-silenced triple-negative breast cancer cell motility (Tosatto et al. 2016). Consistent with previous research, our study showed that HIF-1α expression was attenuated in MCU-silenced HCT-116 and SW-480 cells, indicating that HIF-1α could be a target of MCU in colon cancer. However, a limitation should be pointed out. Unlike other studies, the high concentration of Yoda1 (100 μM) was used in this study, partly due to low sensitivity of HCT-116 and SW-480 cells to Yoda1. However, our results showed that 100 μM Yoda1 could significantly activate the expression of Piezo1 in the two colon cancer cells, which might reflect the heterogeneity between different cancer cells.

Combining with previous research, our findings confirmed that Piezo1 is involved in colon cancer cell metastasis and proposed a potential Piezo1-mediated molecular signaling pathway, Piezo1-MCU-HIF-1α-VEGF.

## Conclusion

Our results suggest that Piezo1 promotes colon cancer cell viability, migration, and metastasis; furthermore, Piezo1 could be involved in a possible regulatory mechanisms of Piezo1-MCU-HIF-1α-VEGF in colon cancer.

## Data Availability

The data sets analyzed during the current study are available from the corresponding author on reasonable request.

## Abbreviations

MCU: Mitochondrial calcium uniporter
HIF-1α: Hypoxia inducible factor 1α
VEGF: Vascular endothelial growth factor
siRNA: Short interfering RNA
qRT-PCR: Quantitative real-time polymerase chain reaction
MMP: Mitochondrial membrane potential
